# Supplementary material for: Are carbon emissions trading and green financial instruments synergistic? -Comprehensive quantitative research based on content analysis
Source: PLoS One. 2024 Mar 7;19(3):e0298601. doi: 10.1371/journal.pone.0298601 (PMC10919601; doi:10.1371/journal.pone.0298601)
Supplement: S1 Data — (ZIP) [file pone.0298601.s001.zip › Data/地级市Z_F工作报告.zip!/║■▒▒/╦μ╓▌╩╨/└┤╫╘╣1⁄2╓┌║┼í╛┬φ┐╦╩2╛▌═°í┐_2018.docx]

各位代表：

　　现在，我代表市人民政府向大会报告工作，请予审议，并请各位政协委员和列席人员提出意见。

一、2017年工作回顾

　　刚刚过去的2017年是不平凡的一年。面对经济新常态下增速换挡、风险防范等多重压力，我们在省委、省政府和市委的坚强领导下，认真贯彻落实党的十八大、十九大精神和习近平新时代中国特色社会主义思想，团结依靠全市人民，以特色提优势、以创新增动能、以实干聚合力，推动经济社会持续健康发展。全市经济企稳回升、稳中有进、进中向好，预计地区生产总值同比增长7%，规模工业增加值增长7.5%，固定资产投资增长16%，地方公共财政预算收入增长9.2%，社会消费品零售总额增长11%，实际利用内联资金、利用外资分别增长10%和9%，城镇、农村常住居民人均可支配收入均增长8%以上。节能减排等控制性指标全面完成省下达目标任务。

　　（一）转型升级重点突破。启动创建经济转型升级和创新驱动示范城市，一手抓传统产业改造提升，一手抓新兴产业培育壮大。强力推进专用汽车及零部件、农产品加工千亿元产业建设，汽车机械产业产值增长25%，呈现近五年最强增势。农产品加工业产值增长15%，创建国家级生态原产地农产品保护示范区通过考评验收。高新技术产业稳步壮大，增加值增长13%。大力实施技改工程，20多家企业“机器换人”，2家企业入选省智能制造试点示范。全面推进市校合作“一园十基地”建设，出台炎帝人才支持计划，与20多所高校精准对接合作。建立大学生创业就业基地45家，吸引大学生实习实训4522人、就业创业3877人。携手武汉理工大学共建工业研究院，新增省级院士专家工作站6个、校企研发中心2家，全市企业创新平台达114家。国家专汽质检中心基本建成，省香菇研究院、风机检测中心投入运营。

　　（二）产业发展协调并进。全力稳固农业基础，粮食总产保持增长，食用菌、畜禽、优质粮油、木本油料、茶叶等产业加快壮大，中粮33万头生猪项目建成投产，随县被授予“中国稻米油之乡”。一二三产业融合发展企业达到210家，获评省示范镇3个。推动工业量质齐升，在石材产业全面关停整顿的背景下，工业用电量增长8.62%，货运量增长20.8%。新能源汽车、智能装备、生物制药和电子信息产业加快发展，新能源产业新增装机69.6万千瓦；建筑业产值突破100亿元。新增产值过10亿元企业4家，齐星集团税收突破3亿元、湖北百强排序上升10位。改造提升现代服务业，金融、旅游、电商等产业蓬勃发展。双星药业挂牌新三板，59家企业登陆四板，设立首期1亿元政府产业引导基金，政府金融平台支持实体经济45亿元，金融机构存贷款余额分别增长12.5%和11.5%。全域旅游加快推进，丁酉年世界华人炎帝故里寻根节成功举办，景区创建力度加大，随州旅游一卡通成功发行，曾都区洛阳镇成为省旅游名镇。全年接待游客2250万人次、旅游综合收入141.1亿元。众志成城投入央视《魅力中国城》竞演，随州荣膺全国十佳魅力城市。

　　（三）项目建设取得成效。召开全市项目建设“比拼赶超”现场会，全年新开工项目2060个，亿元以上项目完成投资490亿元、增长16.5%。首座500千伏智能变电站投产送电，随孝电网解环工程完工，广水电网并入随州。中广核万店、齐星龙岗等地面光伏电站并网发电，金龙新型地板、程力5万辆轻卡、广固数控加工机床、泰华科技二期扩能等项目建成投产，宁西铁路增建二线随县段顺利竣工。汉十高铁、鄂北水资源配置工程随州段、武汉城市圈环线高速广水段等项目推进顺利。拓宽项目融资渠道，9个PPP项目成功落地，发行城市停车场等债券22.4亿元、首笔PPN债券15亿元，获批国开行项目授信300亿元。

　　（四）改革开放扎实推进。供给侧结构性改革落地落实，累计为市场主体降低各类成本25.98亿元，企业亏损面、资产负债率分别下降15.8个和2.9个百分点。“放管服”改革有效推进，市直部门行政职权事项减少23.4%，政府投资核准事项减少30%。实施“29证合一”，新登记市场主体超过3万户。新增混合所有制企业5家。“两室联建、医养结合”模式入选国家养老服务业发展典型案例，农村“两权”抵押贷款试点、不动产统一登记、供销社混合制试点、国有林场等各项改革推进顺利。对外开放取得成果，引进亿元以上项目62个，常青机械、华昶科技、万达广场等成功签约。外贸出口增长8%，其中香菇出口6.5亿美元，居全国地级市首位。成功引进农产品跨境电商平台。

　　（五）城乡面貌明显改观。科学优化区域布局，城乡总体规划在全省率先完成编制审批，成为“湖北样本”。持续增强城市综合承载力，中心城区实施重点项目93项，总投资超过100亿元，胜利大道、清河东路、绕城南路等建成使用，明珠东路、汉东东路和 水一桥、水一桥改造等项目加速推进，桃园大桥启动建设。东、西护城河等黑臭水体治理取得阶段性成效，淅河、城北污水处理厂基本建成。烈山、蒋家岗、水3个农贸市场相继新建，市农副产品物流园二期建成使用。随州欢乐世界游乐场投入营业。成立市城管执法委员会，开展城市环境综合整治“雷霆行动”“秋风行动”，城市管理更加精细。随县新县城路网逐步拉开，烈山湖桥等工程加快实施；广水市印台山文化生态园、广悟大道等项目推进有力，高标准承接了“空降排—2017”国际军事比赛。着力打造美丽村镇，长岗菩提小镇等一批特色小（城）镇建设全面启动，黄木淌村、桃源村分别获评全国美丽宜居村庄、中国乡村旅游创客示范基地。

（六）生态优势不断彰显。全面推进城乡绿色革命，加快“一轴一环三片”生态布局，随城山国家生态公园二期、擂鼓墩遗址公园等一批城市公园游园建成使用，中心城区新增绿地84.5万平方米，曾都区“两大片两条线”生态走廊、随北百里风光走廊、广水北三镇生态画廊、高新区美丽乡村示范片建设成果显现。全市绿满行动造林33.6万亩，森林覆盖率和蓄积量稳步增长，完成天然林落界373.9万亩，林木采伐控制在省定限额10%以内，成功创建省级2个森林城镇、69个绿色示范乡村。37个乡镇生活污水处理厂启动建设，我市全域通过省农村生活垃圾治理考评验收。推行落实河湖长制，水库养殖网箱全部拆除，禁养区28家养殖场全部搬迁，非禁养区77家养殖场治理全部达标。深化大气、水、土壤污染防治，狠抓秸秆禁烧，淘汰黄标车2891辆，空气优良天数比例和PM10、PM2.5浓度值实现“一升两降”，县级以上城市饮用水源地、出境断面水质平均达标率100%，中央、省环保督查问题整改较好完成。

　　（七）人民生活持续改善。财政用于民生领域的支出121亿元，占公共预算支出的80.1%，增长5.3%。打响精准扶贫攻坚战，实施易地扶贫搬迁5998户、12415人，随州扶贫云平台上线使用，83兆瓦光伏扶贫项目建成并网，4.4万贫困人口饮水安全问题得到解决，省定年度脱贫目标圆满完成。稳定扩大就业，增发创业担保贷款近8000万元，城镇新增就业2.16万人。加强社会保障体系建设，推进全民参保登记计划，统一城乡居民医保政策，跨省异地就医实现直接结算，农民工工资支付保障办法出台实施。发放各类社会救助资金和优待抚恤金3.88亿元。住房公积金扩面7800人，发放贷款和提取资金15亿元。保障性住房、棚户区改造住房新开工7848套、基本建成5573套，改造农村危房4488户。重视提升教育办学质量，推进素质教育，普通高中毕业生升学率达到92.1%，高考成绩取得新突破；新建、改扩建校舍5.1万平方米，发放贫困生资助金4200多万元，高新区胜利学校、随县炎帝学校建成使用。大力发展文体事业，完成扩建市博物馆，成功举办市第二届运动会，新建农村文体广场132个，全民阅读、全民健身氛围日渐浓厚，曾都区获评全国群众体育先进单位。健全医疗卫生服务体系，城市公立医院改革顺利启动，市中心医院成功创建三甲、新院区基本建成，市残疾人康复中心完成主体工程。省文化科技卫生“三下乡”集中示范活动在广水市举行。加强和创新社会治理，“三民工程”深入开展。加大平安建设力度，安全生产形势稳定好转，信访维稳、打击犯罪、食药监管、应急管理等工作扎实推进，我市连续三年被授予全省社会治安综合治理优胜单位，社会大局和谐稳定。精神文明建设成果丰硕，连续四届荣获省级文明城市称号，全国文明单位、文明村镇、文明校园达到14个，7人被评为“荆楚楷模”。加强国防动员建设，驻随部队勇担急难险重任务，有力推动了军民融合发展。巩固国家双拥模范城创建成果，优抚安置工作较好落实。

　　（八）政府自身建设得到加强。深入推进全面从严治党，常态化开展“两学一做”学习教育，规范开展“主题党日”活动。自觉接受人大及其常委会法律监督、政协民主监督、社会监督和舆论监督，认真办理人大建议143件、政协提案165件。扎实推进法治政府建设，落实我市首部实体法，城区禁鞭成为常态。加强勤政廉政建设，强化履职尽责清单管理，深化行政效率不高、执法不公“两不”专项整治，开展扶贫领域电视问政，“作风聚焦”暗访曝光不担当、不作为等问题85个、处理146人，治理了一批基层“微腐败”，营造了为民务实清廉的政务环境。

　　与此同时，新闻出版、文联、社科联、外事侨务、对台、民族宗教、援疆和国防教育动员工作再上新台阶，工青妇、儿童、残疾人、红十字会、无偿献血、老龄和慈善事业有了新发展，审计、统计、国调、质监、物价、机关事务、公共资源交易管理、档案、保密和地方志工作迈出新步伐，邮政、通讯、无线电、石油石化、烟草、民防、消防、水文和气象等工作都取得新成绩！

　　各位代表！一年来的发展成就来之不易，这是省委、省政府和市委坚强领导、科学决策的结果，是市人大、市政协监督支持的结果，是历届政府砥砺奋进的结果，是广大党员干部和人民群众携手共进的结果!在此，我代表市人民政府向全市人民，向各位人大代表、政协委员，向各民主党派、工商联、人民团体和无党派人士，向驻随部队、武警官兵，向所有关心、支持、参与随州改革发展的各界朋友，致以诚挚的敬意和衷心的感谢!

　　各位代表！我们也清醒认识到，当前全市经济虽然持续增长，但部分指标增速放缓，地区生产总值、规模工业增加值未实现年初预期目标；实体经济困难较多，特别是信贷投放不足，融资瓶颈仍较突出；脱贫攻坚、环境改善、社会治理等难度增大，教育、医疗等公共服务资源供给不足，交通拥堵等“城市病”治理仍需综合施策。此外，政府自身建设方面，少数部门和工作人员担当不够、履职不力，“四风”问题特别是形式主义、官僚主义出现新表现，表态多调门高、行动少落实差等问题一定程度存在，提高行政质效还需向深度用力。对此，我们一定高度重视，着力加以解决。

二、面临的形势和任务

　　2018年是贯彻党的十九大精神的开局之年，是改革开放40周年，是决胜全面建成小康社会、实施“十三五”规划承上启下的关键一年。站在新的历史起点上，必须观大势、谋全局，找准坐标，顺势而为，奋力开辟新时代随州发展新境界。当前，全球经济复苏乏力，不稳定不确定因素明显增多；我国区域发展呈现新动向，竞争更趋激烈。我市发展不平衡、不充分和规模小、空间小的最大困扰还没有根本改变，做大总量与转型升级、财政增收与企业减负、保护生态与加快发展等两难矛盾增多。同时更要看到，我国经济已由高速增长阶段转向高质量发展阶段，各种积极因素不断积聚。特别是党的十九大胜利召开，描绘了新时代中国特色社会主义的宏伟蓝图。省第十一次党代会作出重大部署，支持随州建设特色产业增长极，省委十一届二次全会吹响了全面建设社会主义现代化强省的号角。随着“一带一路”建设、大别山革命老区振兴发展等重大战略纵深实施，我们凭借深厚的文化底蕴、良好的生态环境、鲜明的产业特色、坚实的项目支撑、优越的交通区位等诸多优势，一定能够抢抓政策窗口期，拓宽发展空间，赢得美好未来！

　　2018年政府工作的总体思路是：全面贯彻落实党的十九大和省委十一届二次全会、市委四届四次全会等精神，以习近平新时代中国特色社会主义思想为指导，按照“五位一体”总体布局、“四个全面”战略布局和湖北现代化强省建设统一部署，坚持以人民为中心的发展思想，坚持稳中求进工作总基调，坚持新发展理念，落实高质量发展的根本要求，狠抓投资拉动、创新驱动、开放带动、实干推动，着力做强实体经济、推动转型升级，着力深化供给侧结构性改革、提高供给体系质量和效益，着力推动产城融合、实施乡村振兴战略、保障和改善民生，深入推进“圣地车都”建设，全力打造特色产业增长极，加快迈向区域性中心城市。

　　今年经济社会发展主要预期目标是：地区生产总值增长8%，规模工业增加值增长8%，固定资产投资增长13%，社会消费品零售总额增长11%，地方公共财政预算收入增长8%，外贸出口增长8%，实际利用内联资金增长10%、利用外资增长8%，城镇、农村居民人均可支配收入增长与经济发展同步，物价形势保持稳定，主要污染物总量减排、环境质量控制等全面完成省下达目标任务。

　　实现上述目标，加快建设富强民主文明和谐美丽随州，我们将坚持“产业兴市、质量强市、创新活市、生态立市”，切实把握以下几点。

　　——坚定不移践行新发展理念。按照创新、协调、绿色、开放、共享的发展思路，以创建经济转型升级和创新驱动示范城市为路径，推动经济发展质量变革、效率变革、动力变革，谋求更加平衡、充分的发展，全面突破“小之困”，努力实现“强省建设”的随州作为。

　　——坚定不移建设现代化经济体系。贯彻落实习近平新时代中国特色社会主义经济思想，坚持质量第一、效益优先、速度合理，调整优化经济结构，夯实专用汽车及零部件、农产品加工、生态文化旅游三极支撑，推动新能源、风机制造、晶体材料、电子信息、智能制造、生物医药、大健康等产业多点突破，加快构建在全省具有比较优势的特色产业格局。

　　——坚定不移推进改革开放创新。坚持改革引领，大胆先行先试，创造更多的随州经验、随州模式。推进全面开放，在广度和深度上下功夫，着力构建开放高地。加强创新型城市建设，完善以企业为主体、市场为导向、产学研深度融合的技术创新体系，不断增强发展新动能。

　　——坚定不移打好三大攻坚战。突出抓重点、补短板、强弱项，坚决打好防范化解重大风险、精准脱贫、污染防治攻坚战，特别是把脱贫攻坚作为政治责任、头等大事和第一民生工程，调动全社会力量，构建大扶贫格局，使全面建成小康社会得到人民认可、经得起历史检验。

　　——坚定不移以人民为中心。着眼满足人民日益增长的美好生活需要，始终把保障和改善民生作为政府工作的出发点和落脚点，一年接着一年干，努力让全市人民幼有所育、学有所教、劳有所得、病有所医、老有所养、住有所居、弱有所扶。

三、2018年重点工作

　　今年政府工作要以党的十九大精神为根本遵循，以推动高质量发展为第一要务，以保障和改善民生为最高追求，重点做好八个方面工作。

　　（一）紧扣转型发展，全力以赴建设特色产业增长极

　　致力打造专用汽车及零部件千亿元产业。加快壮大专汽产业集群，力争产值增长15%以上。依托30公里专汽长廊，引导资源要素聚集，规划建设智能专用车、应急装备等产业园。积极应用新材料、新工艺、车联网等技术，大力研发新能源、轻量化、智能化“高精尖”专汽产品，加快发展应急救援、特种消防、高铁维护等专用车。做大做强整车企业，带动零部件产业配套发展。支持国家专汽质检中心、省专汽研究院发挥功能，不断提升设计、研发、营销等平台服务水平。强化规范整顿和行业自律，提升产品质量和品牌形象，让中国专用汽车之都逐步享誉世界。

　　致力打造农产品加工千亿元产业。深化农业供给侧结构性改革，打造“来自炎帝故里的馈赠”系列农产品区域品牌，积极创建国家级农产品（食用菌）优势区、农业产业化示范基地，加快建设现代化农业强市。拓展农业产业链条，促进种养加、产供销一体化发展，力争农产品加工产值增长10%以上。着力扶持市场主体，支持龙头企业做精做专，培育一批农业产业化联合体。加快发展农产品订单直销、连锁配送，推进鄂北最大的特色农产品加工物流中心建设。

　　致力打造生态文化旅游战略性支柱产业。以“旅游+、+旅游”为方向，加快建设世界华人谒祖圣地、全国知名的旅游目的地。积极培育寻根游、生态游、研学游、工业游等旅游新业态，大力营销推介精品旅游线路，加快开发旅游商品。推进炎帝故里文化旅游区、大洪山、千年银杏谷、桃源村、田王寨等景区创建，力争随县、长岗镇建成省级全域旅游示范区。

　　致力打造成长性新兴产业集群。坚持无中生有、有中生优、优中生强，推动互联网、大数据、人工智能和实体经济深度融合，引导发展互联网教育、移动医疗、共享经济等新业态。积极推进新能源产业发展，谋划实施光伏、生物质发电项目。支持随县通用航空项目一期建设，培育发展航空运输、维修、培训等临空产业。有序扩大中药材种植面积，推进叶开泰智能制药等项目建设，加快发展中成药制造业，引导产业向大健康领域发力。

　　（二）紧扣提质增效，全力以赴做强实体经济

　　加快壮大工业支柱。大力发展先进制造业，优化升级风机制造、晶体材料、电子信息、纺织服装等产业。重点推动犇星化工、东合新能源汽车、常森汽配、金龙复合材料、导航陀螺仪等项目建设。抓好中小企业成长工程，力争新增规模企业50家以上，支持企业争做细分行业“隐形冠军”。深入开展“五个一”包保、全员下沉、“一企一策”、“减负晾晒”等“四大帮扶行动”，破解企业发展难题。实施创新驱动发展战略，滚动推进“百企百亿技改工程”，实施重大技改项目50项以上。鼓励原始创新，引导企业加大研发投入，支持各类创新平台建设，强化科技攻关，推动科技成果转化，力争发明专利增加10%，高新技术产业增加值增长13%以上。

　　加快发展现代农业。夯实农业生产能力基础，稳定粮食种植面积，壮大特色种养业，确保食用菌生产稳定在3亿袋左右，扶持推广香菇集中制棒、分散出菇新模式，力争规模达到1000万袋，惠及菇农5000户以上，支持随县争创国家现代农业（香菇）产业园。加快建设优质粮、蔬菜、木本油料、茶叶、蓝莓、花卉苗木等规模化生产基地，重点发展肉鸡、生猪、肉牛三大畜禽业和小龙虾、花白鲢两大水产养殖业。稳妥推进适度规模经营，发展新型农业经营主体300家以上。狠抓农产品标准化生产，落实质量安全追溯制度。严格耕地保护，加强高标准农田和水利工程建设，强化水库汛情在线监测。探索一二三产业融合发展新路径，鼓励企业发行农村产业融合发展专项债和增信集合债；实施“百县千乡万村”工程，创建17个省级融合发展示范村。

　　加快培育现代服务业。积极适应消费趋势新变化，大力发展全域旅游，力争全年接待游客2400万人次，综合收入突破150亿元。拓展商贸服务业，畅通城乡流通网络，引进大型卖场和商业综合体，推动实体零售创新转型。加快发展电子商务，促进线上线下融合发展。引导发展餐饮、住宿、家政、健康等生活性服务业，培育壮大现代物流、融资租赁、信息技术、节能环保、服务外包、人力资源等生产性服务业。打击侵权假冒，保障消费安全。

　　加快提升县域经济。深入开展“比拼赶超”活动，推动县域经济争先进位。重点支持随县发展农产品加工和全域旅游，广水市发展风机制造和智能装备；曾都区市区一体联动发展，打造专用汽车及零部件特色产业发展主阵地；随州高新区产业升级、产城融合，成为市域经济第一增长极；大洪山提升核心景区，建好佛教名山、养生天堂。大力推进各级各类开发区和工业园区建设，提高配套服务水平。

　　（三）紧扣筑牢底盘，全力以赴扩大有效投资

　　突出项目招引，增强后劲支撑。把招商引资作为“一号工程”，选聘招商顾问，完善招商信息，充分发挥产业招商分局的作用，选准方向、优化布局，推进产业招商、以商招商，探索中介招商，切实提高资金到位率和项目落地开工率。推动央企、省企与我市企业合作重组，扩大混合所有制经济发展成果。开展安商暖商利商行动，落实优惠政策，强化督办考核，力争新引进亿元以上项目40个以上，实际利用内联资金320亿元以上，利用外资1.5亿美元以上。

　　强化项目谋划，优化投资结构。紧盯国家政策导向、投资方向，围绕产业转型升级、基础设施、社会事业、生态保护、乡村振兴等五大领域，超前谋划一批事关长远发展的大项目、好项目，争取更多项目纳入国家和省投资计划。做实随州至信阳高速公路、城南国际物流城等项目前期工作，争取尽早落地开工。

　　推进项目实施，促进增产达效。重点推进汉十高铁、鄂北水资源配置二期、水生态综合治理等重大工程建设，确保程力二期、毅兴智能、双剑风机、正大熟食等产业项目竣工投产。激发民间投资活力，大力实施绕城南路地下综合管廊等PPP项目，加快南北外环一级公路等骨干路网建设，基本建成浪河至何店一级公路。全力推进220千伏江头店、110千伏滨湖变电站等电网配套项目建设。

　　健全项目机制，保障要素供给。完善投资项目在线审批监管平台。继续强化领导包保、五星管理、集中开工、现场观摩、跟踪督办等工作机制，推广曾都区周六现场办公制，定期会商协调解决项目建设难题。下大力气整治施工环境，高效推进融资、用地等要素保障。

　　（四）紧扣全面小康，全力以赴决战脱贫攻坚

　　在协同推进上强责任。围绕实现24个贫困村脱贫出列、3.7万贫困人口脱贫销号的目标，强化各级各部门及“一把手”主体责任，发挥驻村工作队和第一书记作用，建立和落实驻村干部督办、召回等制度。加强扶贫绩效考核评估和监督执纪问责，严把脱贫退出的标准关、程序关、群众认可关，提升“三率一度”，确保脱贫质量，既不降低标准，也不吊高胃口。加强扶贫信息系统建设，整合中国社会扶贫网和随州扶贫云平台，推动政府、市场、社会“三位一体”大扶贫。

　　在产业扶贫上出实招。坚持扶贫同扶志、扶智相结合，引导贫困群众劳动增收、就业创业致富，避免养“懒汉”。推进特色产业扶贫，因地制宜发展种养业，壮大贫困村集体经济。推广“政府、银行、保险、合作社+贫困户”的“四帮一”产业扶贫模式，实施电商扶贫、旅游扶贫等精准扶贫工程。加大各级财政扶贫专项投入，整合项目资金，规范管理使用。加强金融帮扶，全面完成省下达小额扶贫贷款目标。

　　在落实政策上不走样。精准落实扶贫政策措施，加快完善贫困村基础设施和基本公共服务，加大深度贫困村扶持力度。推进农村危房改造，着力解决贫困人口安全饮水问题。做好易地扶贫搬迁后续工作，完成拆旧建新任务，确保稳得住、可脱贫。落实教育、健康和社保兜底政策，确保贫困人口住院费报销比例达到90%以上，个人自付费不超过5000元，有效避免因病返贫；全面落实贫困家庭子女义务教育“两免一补”政策，防止因贫失学。探索资产收益和生态补偿扶贫等途径。

　　（五）紧扣动能转换，全力以赴推进改革开放

　　深化供给侧结构性改革。聚力“五大任务”，聚焦“破、立、降”，大力破除无效供给，以创新转型增强新动能。全面落实减税降费政策，力争为企业再减负2亿元以上，激发各类市场主体活力。同时，加快补齐基础设施、公共服务、民生保障等领域的短板。

　　深化“放管服”改革。持续转变政府职能，加大“互联网+放管服”改革力度，推进神农云建设，促进数据通、业务通，尽快实现“一网覆盖、一次办好”目标，力争让群众企业办事像网购一样方便。坚持放权到位、管理增效、服务提质，推广政务服务APP，加快网上审批服务向基层延伸，开展争创“全省最快办件”活动。巩固扩大“多证合一”商事制度改革成果，推进企业登记全程电子化，深化高新区“一窗受理、组团服务”改革，全面提高行政服务效率。推进综合执法体制改革，加大市场监管力度。

　　深化重点领域改革。推进预算管理制度改革，优化支出结构，提高财政资金使用效益。拓宽社会融资渠道，大力发展普惠金融，推动汉口银行等金融机构进驻随州，力争全年新增社会融资规模120亿元。积极对接多层次资本市场，引导企业主板上市，力争挂牌新三板、四板企业分别新增1家和10家以上，推动更多企业运用债券融资工具。大力发展股权融资，利用产业引导基金，有效撬动社会资本。创新政府增信模式，支持融资性担保机构发展。树立与风险赛跑的意识，关口前移、分类施策，稳妥处置政府债务、非法集资等风险，做好隐性债务化解工作。推进统计体制改革，做好第四次全国经济普查工作。加快国有企业公司制改革，做好“三供一业”分离移交。

　　深化对外开放合作。完善随州口岸功能，加强大通关建设，争取设立公用型保税物流中心。加快“走出去”步伐，积极对接“一带一路”和湖北自贸区建设，不断扩大专用汽车、食用菌、茶叶、通讯电子等产品出口份额。充分利用国际国内两个市场两种资源，引导企业到境外投资发展。深度融入长江经济带等战略，探索区域合作新路径，加强产业对接，促进共赢发展。

　　（六）紧扣生态宜居，全力以赴建设美丽随州

　　抓扩容提质，让城市更有品位、管理科学精细。发挥规划引领作用，统筹城乡一体化发展，推进国家新型城镇化综合试点建设。围绕拉开框架、完善功能，投资112亿元，建设中心城区重点项目77项。完善城南和淅河片区路网，新建明珠东路、白云大道北段等城市道路，改造汉东路中段、烈山大道中段等主干道。加快市委党校新校园、城市公共活动中心综合体、随州中心客运站、大隋文化创意产业园等项目建设。完善城市便民市场，改造老旧零售菜市场，新建草店子街净菜市场。围绕打造特色产业增长极的核心区、创新转型的先行区、现代服务业的引领区、高端人才的集聚区和宜居宜业宜游的示范区，统筹谋划城南新区高铁新城建设。启动4平方公里的高铁小镇建设，快速推进高铁站房、站前广场、站前大道、停车场、综合管廊等基础设施配套，实现与高铁通车无缝对接；推动产城融合，加大招商力度，重点发展会展博览、研发设计、电子商务、现代物流、文化创意、健康养老等产业，放大高铁枢纽优势；加快承接老城区疏解的行政及事业性服务机构、批发市场、较大物流节点等功能。进一步创新完善城市管理体制，发挥数字城管平台功能，严查违法建设、占道经营、渣土漏撒、广告杂乱和乱搭乱建等行为，巩固环卫作业市场化改革成果。推动随县和广水市城市基础设施提档升级，提高精细化、规范化、数字化管理水平。

　　抓乡村振兴，让农村山清水秀、农民安居乐业。按照产业兴旺、生态宜居、乡风文明、治理有效、生活富裕的总要求，建立健全城乡融合发展体制机制和政策体系，大力实施乡村振兴战略。全面开展“三乡工程”，促进市民下乡、能人回乡、企业兴乡，推动城市资金、技术、人才、信息等要素与农村资源有效对接。加强支农资金整合使用，鼓励社会力量参与农村建设。积极培育长岗菩提小镇、洪山温泉康养小镇、应办多肉艺术小镇、洛阳银杏小镇等特色小镇，建好一批共享田园综合体试点，带动产业增效、农民增收、生态增值。探索“三权分置”多种实现形式，推进农村集体产权制度改革，进一步盘活土地资源。加快提升农村基础设施，改善道路、供水、供电、通讯等生产生活条件，新建通村公路350公里、2处公交首末站。启动开展“厕所革命”三年攻坚行动，统筹推进城市、旅游景点尤其是农村厕所建设，切实让群众“方便”更方便。深化农村环境综合整治，确保生活垃圾统筹治理全覆盖。进一步推广“荆楚派”特色民居示范建设，创建一批国家级、省级宜居村庄。

　　抓绿色发展，让自然宁静和谐、环境持续改善。践行绿水青山就是金山银山的理念，推进绿色低碳循环发展。实施“蓝天工程”，持续开展“两禁、三控、四治”，完善大气污染防治考核办法，让随州呈现更多高颜值的蓝天白云。实施“碧水工程”，全面落实河湖长制，实行“一河一策”。加强水环境综合治理，开展封江口水库饮用水源保护区环境整治，强化全市34个乡镇集中式饮用水源地保护。实施“净土工程”，启动土壤污染详查，制定实施污染治理与修复规划，继续推进矿山复绿。加强农业面源污染防治，推进畜禽养殖废弃物资源化利用。实施“增绿工程”，深入推进“一轴一环三片”建设，加快涢水、漂水、花溪河湿地公园建设，确保中心城区新增绿地60万平方米。继续封山育林，大力开展精准灭荒，强化天然林、生态公益林、古树名木等管护，发展林下特色经济，实施林业碳汇工程，壮大绿色富民产业。大力推进森林城镇和绿色示范乡村创建，加快淮河、徐家河国家湿地公园试点建设，争创封江口国家湿地公园、中华山鸟类国家级自然保护区。

　　（七）紧扣普惠发展，全力以赴谋求民生福祉

　　着力提升公共服务。优先发展教育事业，坚持立德树人，发展素质教育。推进城乡义务教育一体化发展，大力缓解“大班额”问题。加快普及高中阶段教育，继续提高办学质量。重视发展学前教育、职业教育、特殊教育等各类教育。深化健康随州建设，加快医药卫生体制改革，重点推进城市公立医院综合改革，落实分级诊疗制度，抓好医疗联合体建设，定向委托培养乡村医生，争创国家卫生城市。加强和完善计划生育服务管理，促进人口长期均衡发展。推进基层食品药品监管机构规范化发展，建立最严格、全过程监管制度，开展小作坊、小餐饮、摊贩“三小”治理，保障人民群众“舌尖上的安全”。继续推进国家“医养结合”试点建设，探索多种模式兴办养老服务示范机构。

　　着力完善多层次社会保障体系。全面实施全民参保计划，加大扩面征缴力度，推进事业单位养老保险制度改革。完成城乡居民医保制度整合“七统一”，扩大参保和支付范围。继续提升社会保障水平，健全城乡低保、农村五保和孤儿生活养育标准自然增长机制，做好城镇困难职工解困脱困工作。大力推进以优抚安置为主体的双拥共建。

　　着力扩大就业创业。落实就业再就业政策，加大就业援助力度，抓好重点群体就业工作。强化技能培训，继续开展“神农工匠”评选。加快创业孵化基地建设，充分发挥创业担保贷款帮扶作用，推进大众创业万众创新，力争新登记市场主体3万户以上。

　　着力强化人才支撑。深入推进人才强市建设，落实炎帝人才支持计划，大力开展“我选湖北·聚才随州”行动。高标准推进“一园十基地”建设，抓好高端人才、创新团队引进和人才培育服务，建好市人才创新创业超市，引导推动创新资源向随州汇聚，努力让各类人才的聪明才智充分涌流、各个领域的创造活力竞相迸发。

　　着力增强文化软实力。挖掘炎帝文化、编钟文化、曾随文化、红色文化等特色地域文化内涵，大力推进文化创造性转化、创新性发展。办好戊戌年寻根节和编钟出土40周年纪念活动，推进编钟音乐文化之都产城融合发展示范区建设。实施中国传统节日振兴工程，丰富传统节日内涵，积极引导、推动大众文化消费。大力弘扬乡土文化，建设新乡贤文化，让随州儿女记得住乡愁。继续推进“三馆一站”免费开放，深入实施全民健身计划，建成一批群众身边的运动场地设施。加强国家历史文化名城保护，推进非物质文化遗产保护传承，创作一批文化精品力作。做好地方志、年鉴编纂出版工作。建立健全精神文明建设长效机制，践行社会主义核心价值观，争创全国文明城市。

　　着力办好民生实事。始终把人民对美好生活的向往作为奋斗目标，坚持尽力而为、量力而行，继续实施一批民生实事项目。（1）促进就业创业，力争城镇新增就业1.6万人，失业人员再就业3000人。发放创业担保贷款7000万元，扶持创业500人，创业带动就业2000人。（2）实施安居工程，全市改造棚户区7228户，公租房分配入住率达到90%以上，其中中心城区改造棚户区5100户；开展小区物业管理专项整治，推进3个以上小区物业服务标准化试点建设。发放住房公积金提取贷款3亿元，支持新市民住房消费。（3）提升中心城区品质，实施2至3个老旧小区提档升级，完成烈山大道中段临街立面包装，改造维修30座城市公厕。（4）改善城镇水环境，提标升级市污水处理厂，基本完成中心城区东护城河改造等黑臭水体治理，确保37个乡镇污水处理厂全部建成营运。（5）继续缓解交通拥堵，建成 水一桥、涢水一桥、实验小学及大润发人行天桥，推进桃园大桥建设，畅通聚玉街、花溪巷等城市微循环；新建5处城市停车场，增加停车位1500个。（6）改善义务教育办学条件，投入5000万元实施“全面改薄”工程；改扩建东城文峰塔和蒋家岗学校，建成高新区云龙小学；新建白云湖初中和南郊擂鼓墩学校，确保2019年秋季开学投入使用；筹措资金4700万元，资助5万人次家庭困难学生。（7）加大文化惠民力度，新建100个乡村、社区文体广场，开展送戏下乡300场、戏曲进校园600场；启动擂鼓墩遗址保护区建设。（8）加强治安防控设施建设，中心城区新增A类公众视频探头200个，建成人像识别系统。

　　（八）紧扣和谐稳定，全力以赴共建共治共享

　　进一步加强社会治理。深化实施“三民工程”，完善社区协商议事机制。加强农村基层基础工作，健全自治、法治、德治相结合的乡村治理体系。规范社会组织管理，大力推进政府购买服务。加快发展志愿服务和慈善事业。加强信用随州建设，健全市场主体信用监管机制，弘扬诚信正能量。推进社会心理服务体系建设，着力培育理性平和的社会心态。

　　进一步强化安全生产。弘扬生命至上、安全第一的思想，严格落实安全生产责任制，加强安全监管执法机构建设，明确乡镇安监机构职责。大力开展安全隐患排查，强化重点行业领域专项整治，深化“打非治违”行动，坚决遏制重特大事故发生。提升防灾减灾救灾能力，加强地质灾害防范和治理。

　　进一步打造平安随州。健全社会矛盾排查预警和调处化解机制，引导群众依法逐级走访，推进信访维稳法治化建设。完善立体化、信息化治安防控体系，依法打击和惩治黄赌毒、黑拐骗等违法犯罪活动。加快推进公共法律服务体系建设，着力保护人身权、财产权、人格权，使人民群众的获得感、幸福感、安全感更加充实、更有保障、更可持续。

四、加强政府自身建设

　　新时代、新使命、新征程，对政府工作提出了新的更高要求。我们将不断增强治理服务能力，以永不懈怠的精神状态和一往无前的奋斗姿态，努力开创各项工作新局面。

　　（一）旗帜鲜明讲政治。切实提高政治站位，牢固树立“四个意识”，增强“四个自信”，坚决维护以习近平同志为核心的党中央权威和集中统一领导。用全面从严治党统领政府工作，落实主体责任和“一岗双责”，严格遵守政治纪律和政治规矩。推进“两学一做”学习教育常态化、制度化，扎实开展“不忘初心、牢记使命”主题教育，用党的创新理论武装头脑、指导实践。

　　（二）依法行政重监督。大力弘扬法治精神，推动各级干部尊法学法守法用法，自觉运用法治思维和法治方式解决问题、化解矛盾、推动工作。自觉接受人大和政协监督，扎实做好建议提案办理工作。广泛听取人民团体意见，主动接受社会和舆论监督。落实重大行政决策程序规定，严格执行“三重一大”制度，拓宽政务公开渠道，让行政权力始终在法治轨道上运行。

　　（三）上下同心聚合力。认真贯彻落实中央大政方针和省委、省政府决策部署，全面执行落实市委决议、决定和统一安排，切实增强全局意识、责任意识、担当意识，杜绝政出多门、各行其是、推诿扯皮，确保政令畅通。整合行政资源，加强团结协作，调动各方面积极因素，“上下同欲对标干、突出重点精准干、撸起袖子加油干、遵循规律科学干、扑下身子务实干”，同心同向、同频共振，形成和衷共济促发展的生动局面。

　　（四）改进作风提效能。大力倡导“亲自、立即、现在、马上”的工作作风，狠抓履职尽责，实行重点工作项目化、清单化管理，全面整肃庸政懒政怠政行为，确保把任务清单变成美好现实。严格落实市委贯彻“八项规定”精神实施办法，深入查找“四风”突出问题，特别是形式主义、官僚主义的新“变种”，采取过硬措施，坚决整改纠偏。坚持严管与厚爱结合、激励与约束并重，探索建立推动高质量发展的工作绩效考评体系，有效激发党员干部干事创业的积极性、主动性和创造性。

　　（五）清正廉洁树形象。深入推进政府系统党风廉政建设，坚持无禁区、全覆盖、零容忍，始终保持反腐高压态势，进一步强化不敢腐的震慑、扎牢不能腐的笼子、增强不想腐的自觉，促进干部清正、政府清廉、政治清明。健全行政权力约束机制，注重巡察结果运用，加强审计等监督。实行公共资源配置全程公开、全程留痕，强化“三公”经费、征地拆迁、民生项目等重点领域的监管，大力整治和查处侵害群众利益的各类“微腐败”，以实际成果取信于民。

　　各位代表！责任呼唤担当，使命引领未来。面对波澜壮阔的新时代，让我们更加紧密地团结在以习近平同志为核心的党中央周围，在省委、省政府和市委的坚强领导下，凝心聚力、锐意进取，勇于担当、真抓实干，奋力谱写决胜全面小康、建设现代化随州的崭新篇章！
